# Benchmarking highly entangled states on a 60-atom analogue quantum simulator

**DOI:** 10.1038/s41586-024-07173-x

**Published:** 2024-03-20

**Authors:** Adam L. Shaw, Zhuo Chen, Joonhee Choi, Daniel K. Mark, Pascal Scholl, Ran Finkelstein, Andreas Elben, Soonwon Choi, Manuel Endres

**Affiliations:** 1 California Institute of Technology, Pasadena, CA USA; 2 Center for Theoretical Physics, Massachusetts Institute of Technology, Cambridge, MA USA; 3 The NSF AI Institute for Artificial Intelligence and Fundamental Interactions, Cambridge, MA USA; 4 Department of Electrical Engineering, Stanford University, Stanford, CA USA

**Keywords:** Quantum simulation, Quantum information

## Abstract

Quantum systems have entered a competitive regime in which classical computers must make approximations to represent highly entangled quantum states^1,2^. However, in this beyond-classically-exact regime, fidelity comparisons between quantum and classical systems have so far been limited to digital quantum devices^2–5^, and it remains unsolved how to estimate the actual entanglement content of experiments^6^. Here, we perform fidelity benchmarking and mixed-state entanglement estimation with a 60-atom analogue Rydberg quantum simulator, reaching a high-entanglement entropy regime in which exact classical simulation becomes impractical. Our benchmarking protocol involves extrapolation from comparisons against an approximate classical algorithm, introduced here, with varying entanglement limits. We then develop and demonstrate an estimator of the experimental mixed-state entanglement^6^, finding our experiment is competitive with state-of-the-art digital quantum devices performing random circuit evolution^2–5^. Finally, we compare the experimental fidelity against that achieved by various approximate classical algorithms, and find that only the algorithm we introduce is able to keep pace with the experiment on the classical hardware we use. Our results enable a new model for evaluating the ability of both analogue and digital quantum devices to generate entanglement in the beyond-classically-exact regime, and highlight the evolving divide between quantum and classical systems.

## Abstract (web summary)

Fidelity benchmarking of an analogue quantum simulator reaches a high-entanglement regime where exact classical simulation of quantum systems becomes impractical, and enables a new method for evaluating the mixed-state entanglement of quantum devices.

## Main

**Fig. 1:**
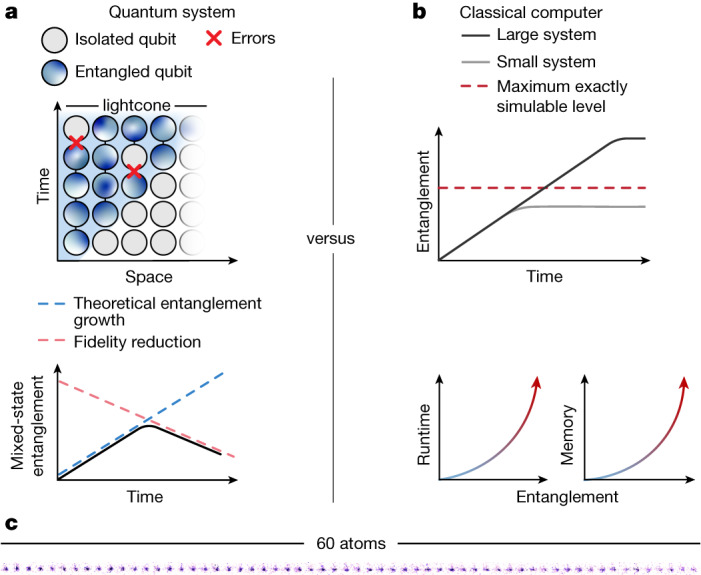
Entanglement in quantum and classical systems. **a**, In quantum systems, entanglement spreads between neighbouring particles before saturating at an extensive level. However, entanglement growth is hampered by experimental errors that reduce the fidelity, limiting entanglement build-up. **b**, On the other hand, classical computers use approximate simulation algorithms that can often only capture a limited degree of entanglement to avoid an exponential increase in cost, meaning they cannot exactly simulate dynamics at large system sizes and long evolution times. **c**, Here we compare quantum devices and classical algorithms in their ability to prepare highly entangled states using a Rydberg quantum simulator with up to 60 atoms in a one-dimensional array (shown as a fluorescence image).

Classical computers generally struggle to exactly represent highly entangled states^7–9^, in the sense of entanglement entropy. This has raised interest in the potential of quantum devices to efficiently solve certain classically hard problems, but modern noisy-intermediate-scale quantum^1,10^ (NISQ) devices are limited by experimental errors (Fig. 1a). This makes it a key goal to benchmark NISQ devices in the highly entangled regime in which exact classical simulation becomes infeasible (Fig. 1b); for example, state-of-the-art classical simulation of Hamiltonian time evolution generating highly entangled states with exact global fidelity is currently limited to 38 qubits (ref. ^11^).

One such approach is to study the fidelity of preparing a highly entangled target state of interest^2^, with several efficient fidelity estimators^12–16^ having been introduced in recent years. However, in the beyond-classically-exact regime, these protocols have only been applied to digital quantum devices, with no such demonstrations on analogue quantum simulators^17^, that is, quantum devices tailored to efficiently encode select problems of interest^18–20^.

In this work, we perform fidelity estimation with an analogue quantum simulator targeting highly entangled states that are impractical to represent exactly on a classical computer. Our Rydberg quantum simulator^14,19^ has recently demonstrated^21^ two-qubit entanglement fidelities of ≈0.999, spurring this study with up to 60 atoms^22^ in a one-dimensional array (Fig. 1c). We stress that we target high entanglement entropy states that require an exponential number of coefficients to represent classically, as distinct from Greenberger–Horne–Zeilinger (GHZ), cluster or stabilizer states, which are efficiently representable on a classical computer at all system sizes^23^ (Supplementary Fig. 1).

Our fidelity estimation is based on extrapolation from benchmarking against many approximate classical simulations, namely, matrix product state (MPS) algorithms that cap the maximum simulation entanglement to avoid the aforementioned exponential increase in classical cost^23–25^ (Fig. 1b). In one-dimension, early-time entanglement growth is system-size independent, so at short times the MPS representation is exact for essentially arbitrarily large systems. When entanglement growth surpasses the entanglement cap, the MPS is no longer a faithful reference, but we can extrapolate the fidelity through a combination of varying the system size, evolution time and simulation entanglement limit.

Using the fidelity, we derive and demonstrate a simple proxy of the experimental mixed-state entanglement^6^, which so far has been notoriously difficult to measure in large systems. Our proxy serves as a universal quality-factor requiring only the fidelity with, and the entanglement of, the ideal target pure state. This enables comparisons between our experiment and state-of-the-art digital quantum devices^2–5,26^, with which we are competitive.

Ultimately, we compare the fidelity of our experiment against that achieved by a variety of approximate classical algorithms, including several not based on MPS. Using a single node of the Caltech central computing cluster, none of the tested algorithms is able to match the experimental fidelity in the high-entanglement regime, except for an improved algorithm we introduce, termed Lightcone-MPS. Even with this new algorithm, classical costs reach a regime requiring high-performance computing to match the experiment’s performance.

## Fidelity estimation with approximate algorithms

A key quantity when studying quantum systems is the fidelity^27^, $F=\langle \psi | {\widehat{\rho }}_{\exp }| \psi \rangle$, where $\left|\psi \right\rangle$ is a pure state of interest and ${\widehat{\rho }}_{\exp }$ is the experimental mixed state. For digital devices studying deep circuits, the fidelity can be estimated by means of the linear cross-entropy^2,12^, a cross-correlation between measurement outcomes of an experiment and an exact classical simulation. A modified cross-entropy, termed^15^ *F*_d_, was proposed for both analogue and digital systems, and demonstrated on Rydberg^14^ and superconducting^28^ analogue quantum simulators. *F*_d_ is efficiently sampled (Supplementary Fig. 15) as

1 $$\begin{array}{r}{F}_{{\rm{d}}}=2\frac{\frac{1}{M}{\sum }_{m=1}^{M}p({z}_{m})/{p}_{{\rm{avg}}}({z}_{m})}{{\sum }_{z}p{(z)}^{2}/{p}_{{\rm{avg}}}(z)}-1,\end{array}$$

where *M* is the number of measurements, *z*_*m*_ is the experimentally measured bitstring, *p*(*z*) is the probability of measuring *z* with no errors following quench evolution and *p*_avg_(*z*) is the time-averaged probability of measuring *z*. *F*_d_ ≈ *F* for a wide class of physical systems, as long as the rescaled probabilities *p*(*z*)/*p*_avg_(*z*) follow the so-called Porter–Thomas distribution^15^. Still, a stringent requirement remains: access to an exact classical simulation to obtain *p*(*z*), precluding direct fidelity estimation at large system sizes. We circumvent this constraint by introducing a method to estimate the fidelity by benchmarking against approximate classical simulations.

**Fig. 2:**
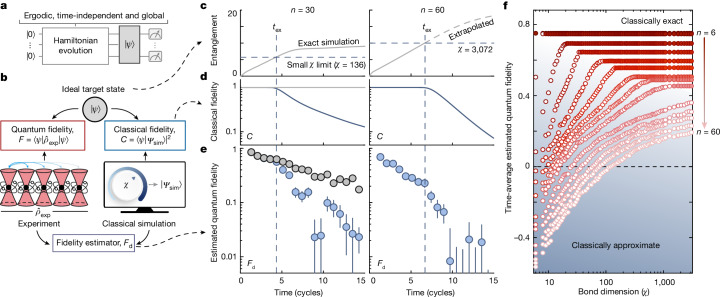
Failure of fidelity estimation with an approximate classical algorithm. **a**, We use a Rydberg quantum simulator and a classical computer to simulate a time-independent, high-temperature quench starting from the all-zero state, targeting an ideal pure state, $\left|\psi \right\rangle$. **b**, The classical algorithm is characterized by a bond dimension, *χ*, which limits the maximum simulable entanglement, resulting in smaller-than-unity classical simulation fidelity, *C*. We estimate the quantum fidelity, *F*, with a cross-correlation between measurement outcomes of the classical and quantum systems, termed^15^ *F*_d_. **c**–**e**. The top shows half-cut von Neumann entanglement entropy of $\left|\psi \right\rangle$, the middle shows classical simulation fidelity, and the bottom shows the estimated experimental quantum fidelity. We study benchmarking against an exact simulation (grey) or an approximate simulation with limited bond dimension (blue). **c**, For a system size of *n* = 30 (left panels), using too small a bond dimension sets a cap in the simulation entanglement. **d**, This causes the classical fidelity to fall at a time, *t*_ex_, when the entanglement of the target state becomes too large. **e**, At roughly *t*_ex_, the estimated experimental quantum fidelity also drops. For the largest system size, *n* = 60 (right panels), *t*_ex_ is well before when the entanglement saturates, even for the largest bond dimension we use. The time-axis is normalized by the Rabi frequency (Supplementary Information). **f**, The estimated fidelity (averaged over all times in **e**) increases with bond dimension (open markers), before saturating (closed markers) at a bond dimension capturing the necessary entanglement. For the largest system sizes, saturation is not achieved using the available classical resources.

We consider a comparison (Fig. 2b) between an ideal high-entanglement target pure state, $\left|\psi \right\rangle$, the experimental mixed state, ${\widehat{\rho }}_{\exp }$, and a pure state from classical MPS simulation, $\left|{\varPsi }_{{\rm{sim}}}\right\rangle$. We introduce an improved MPS time-evolution algorithm using an optimal decomposition of Hamiltonian dynamics into quantum circuits^29,30^, which we term Lightcone-MPS (Supplementary Information). The MPS is parameterized by a bond dimension, *χ*, that defines the maximum simulable entanglement, which scales as $\log (\chi )$. Starting from an all-zero state, we program a time-independent, global quench under the one-dimensional Ising-like Rydberg Hamiltonian (Fig. 2a, for Hamiltonian details see Supplementary Fig. 3 and the Supplementary Information). Hamiltonian parameters lead to high-temperature thermalization^31^, such that describing $\left|\psi \right\rangle$ at late times requires an exponential number of classical coefficients^14^.

For a system size of *n* = 30 (Fig. 2c–e, left), we can exactly classically simulate these dynamics (Fig. 2d, grey); by exact, we mean the classical fidelity, $C=| \langle {\varPsi }_{{\rm{sim}}}| \psi \rangle {| }^{2}$, stays near unity for all times. We numerically observe the entanglement of the target state increases linearly at early times, before eventual near-saturation (Fig. 2c). Moreover, the estimated experimental quantum fidelity, *F*_d_, shows apparent exponential decay due to experimental errors^14^ (Fig. 2e, grey).

However, the situation changes when using an approximate classical simulation. Now, the classical fidelity begins to decay (Fig. 2d, blue) after the time, *t*_ex_, when the ideal entanglement exceeds the limit set by the bond dimension (Fig. 2c, blue), meaning the classical simulation is no longer a faithful reference of the ideal dynamics. Most importantly, we find that after *t*_ex_ the experimental benchmarked fidelity also deviates downwards (Fig. 2e, blue), indicating that *F*_d_ no longer accurately estimates the fidelity to the ideal state. For the largest system sizes (for instance, *n* = 60 in Fig. 2c–e, right), *t*_ex_ occurs well before the entanglement is predicted to saturate, even for the largest bond dimension we can realistically use. We estimate the classical fidelity in this case using the product of MPS truncation errors^25^, which we find is accurate in the regime in which we operate (Supplementary Fig. 29).

Essentially, *F*_d_ seems to be an amalgam of both classical and quantum fidelities, only estimating the quantum fidelity to the ideal state in the limit of the classical simulation being perfect. To test this behaviour for all system sizes, we study the benchmarked value of *F*_d_ averaged over all experimental times (Fig. 2f). Consistently, we see for a bond dimension (open markers) that is too small, *F*_d_ is reduced. In some cases, the requirement that *p*(*z*)/*p*_avg_(*z*) follows a Porter–Thomas distribution can be violated, resulting in *F*_d_ even becoming unphysically negative. As bond dimension increases, *F*_d_ rises, before reaching a saturation bond dimension, *χ*_0_(*n*, *t*), which depends on system size and time (closed markers). For the largest system sizes and times, however, the saturation bond dimension is beyond the capabilities of any current classical hardware^11^.

If the noise affecting the system was purely Markovian, then the fidelity would decay exponentially^32^ and it would be possible to measure the fidelity at early times before *t*_ex_ to learn the exponential decay rate, and then extrapolate in time to estimate the late-time fidelity. Indeed, we note this is an advantage of the *F*_d_ metric we use here, because it accurately estimates the fidelity earlier than other estimators such as the standard linear cross-entropy^14,15^. However, extrapolating to late times is non-trivial in our case owing to non-Markovian noise sources often affecting analogue quantum systems. In particular, with analytic and numerical analysis we show that shot-to-shot Hamiltonian parameter fluctuations (for example, laser intensity variations) induce subexponential fidelity decay at low fidelities (Supplementary Information Theorem 1 and Supplementary Fig. 8).

**Fig. 3:**
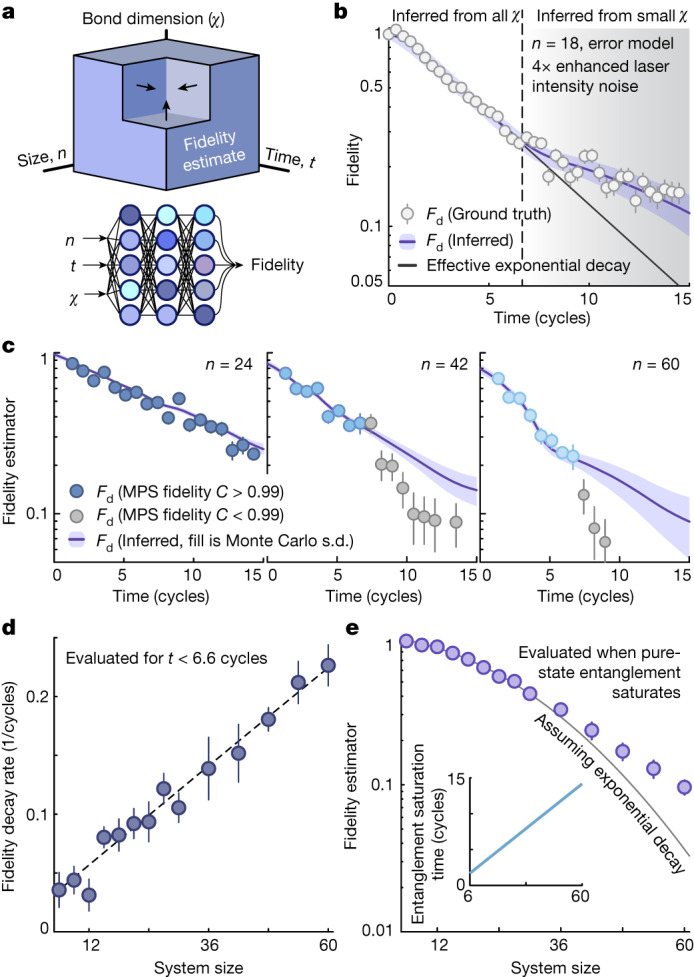
Fidelity benchmarking a 60-atom system. **a**, We use a Monte Carlo inference approach to extrapolate the fidelity at large system sizes and long evolution times. Specifically, we train 1,500 neural networks, each instantiated with randomized (hyper)parameters, to predict *F*_d_ as a function of size, time and bond dimension, and take the ensemble average as the predicted value. **b**, We test this procedure using error model simulations from *n* = 8 to 18 with increased laser intensity noise to emulate the fidelity expected for the experimental *n* = 60 dataset. For *t* > 6.6 cycles and *n* > 15, we only train on bond dimensions below the level necessary for exact simulation to mimic constraints at large system sizes. We observe two behaviours: (1) the ensemble prediction is consistent with the ground truth, and (2) the fidelity seems to follow a non-exponential form. See the Supplementary Information for further cross-checks, as well as analytic evidence for the origin of the non-exponential decay due to non-Markovian noise. **c**, Experimental fidelities for *n* up to 60; markers are grayscale where the classical fidelity (with *χ* = 3,072) is less than 0.99. **d**, Early-time fidelity decay rate as a function of system size, consistent with linear system-size scaling. **e**, Fidelity at the time (inset) at which the pure state entanglement saturates, with *F*_d_ = 0.095(11) at *n* = 60; the error bar is the standard error over Monte Carlo inferences added in quadrature with the underlying sampling error.

Instead, we use a model-agnostic extrapolation by leveraging a large amount of data with three independent parameters: evolution time, system size and bond dimension normalized by its saturation value (Fig. 3a and Supplementary Information). We can calculate *F*_d_ in seven of the octants of this parameter space: the only outlier is the high-entanglement regime of interest. We thus use a Monte Carlo inference approach by training an ensemble^33^ of initially randomized neural networks to predict *F*_d_ given an input *n*, *χ* and *t*; *F*_d_ at large system sizes and long evolution times is then estimated as the ensemble average when *χ* → *χ*_0_ (Supplementary Fig. 9). We emphasize that essentially we are simply performing curve fitting of the smoothly varying function *F*_d_(*n*, *χ*, *t*), for which we can directly simulate many ground truth data.

We check that this protocol consistently reproduces fidelities at small system sizes, does not seem to overfit the experiment (Supplementary Fig. 12), is insensitive to hyperparameters such as the neural net topology and size, and that predictions are converged as a function of the bond dimension (Supplementary Fig. 13). We further reaffirm that our method extrapolates correctly by replicating our entire procedure in a smaller scale wherein the quantum device is replaced by numerical error model simulations up to *n* = 18 atoms (Supplementary Information). For *t* > 6.6 cycles and *n* > 15, the training data only consist of low bond dimensions to emulate the limitations of the large-*n* experimental data. Even still, the extrapolated fidelity is in excellent agreement with the ground truth data (Fig. 3b and Supplementary Fig. 12), and reproduces the subexponential fidelity decay predicted analytically (Supplementary Information Theorem 1).

Ultimately, we apply Monte Carlo inference to the full experimental dataset for system sizes up to *n* = 60 atoms (Fig. 3c; see Supplementary Figs. 7 and 11 for all data). At high fidelities (roughly greater than 0.2), we observe nearly exponential decay, with a rate scaling linearly with system size (Fig. 3d). At low fidelity, however, the Monte Carlo prediction again reproduces the expected subexponential response. We estimate the fidelity to produce the target state when the entanglement is expected to saturate (Fig. 3e), yielding *F*_d_ = 0.095(11) at *n* = 60.

 This work showcases benchmarking a quantum device by extrapolating from approximate classical simulations, and extends the reach of global fidelity estimation for analogue quantum simulators into the classically inexact regime. We expect this approach to be scalable; by studying the convergence of predicted fidelities as a function of bond dimension, our approach seems feasible for up to an order-of-magnitude more atoms than we use here (Supplementary Fig. 14).

## Experimental mixed-state entanglement

Having benchmarked the fidelity of our Rydberg quantum simulator, we now turn to investigate the actual half-chain bipartite entanglement content of the experiment. In the past, several studies have investigated entanglement properties of (nearly) pure states by estimating the second Rényi entropy in (sub)systems up to ten particles^31,34–36^. However, the actual output of an experiment can be a highly mixed state with markedly different entanglement content from the target pure state. For this reason, it is desirable to directly quantify mixed-state entanglement measures. Unfortunately, extensions of most pure state entanglement measures to the case of mixed states are defined variationally, and as such are incalculable for even moderately sized systems^37^.

An alternative, computable measure of mixed-state entanglement is the log negativity^6^, ${{\mathcal{E}}}_{N}$, which is an upper bound to the distillable entanglement of the system^37^. However, measuring the value of the negativity naïvely requires tomography of the full system density matrix, which is infeasible even for intermediate scale quantum systems^38,39^. In the past, experiments have been limited to demonstrating necessary conditions for a non-vanishing negativity, which can only reveal the binary presence of mixed-state entanglement^40,41^.

Here we derive and demonstrate an entanglement proxy, ${{\mathcal{E}}}_{P}$, which can lower-bound the extensive mixed-state entanglement (quantified by log negativity). For a mixed state, $\widehat{\rho }$, with fidelity, *F*, to a target pure state, $\left|\psi \right\rangle$, with known entanglement, ${{\mathcal{E}}}_{N}(\left|\psi \right\rangle )$, our mixed-state entanglement proxy is

2 $$\begin{array}{r}{{\mathcal{E}}}_{P}(\widehat{\rho })\equiv {{\mathcal{E}}}_{N}(\left|\psi \right\rangle )+{\log }_{2}(F).\end{array}$$

Here, ${{\mathcal{E}}}_{P}$ is a proxy evaluating the competition between the growth of the error-free entanglement, ${{\mathcal{E}}}_{N}(\left|\psi \right\rangle )$, versus the error-sensitive fidelity, as *F* < 1 reduces the mixed-state entanglement. When $\widehat{\rho }$ is an isotropic state (an admixture of a maximally entangled state and a maximally mixed state), it has been shown^6,42^ that ${{\mathcal{E}}}_{N}(\widehat{\rho })=\max ({{\mathcal{E}}}_{P}(\widehat{\rho }),0)$ at large system sizes. Further, we show the same holds for a Haar-random state admixed with a maximally mixed state—the expected output^32^ of deep noisy random unitary circuits (RUCs)—as long as the fidelity is large compared to the inverse of the half-chain Hilbert space dimension (Supplementary Information).

More generally, we prove ${{\mathcal{E}}}_{P}$ is a lower bound for ${{\mathcal{E}}}_{N}$ for any mixed state assuming $\left|\psi \right\rangle$ is the highest fidelity state to $\widehat{\rho }$, and becomes tighter as the system size increases (Supplementary Fig. 17). Violations of this assumption can only lead to small violations of our bound in the worst case for physically realistic conditions with local or quasi-static errors, as we show with both analytic (Supplementary Information Theorems 3 and 4) and numeric (Supplementary Figs. 18 and 21) support in the Supplementary Information.

**Fig. 4:**
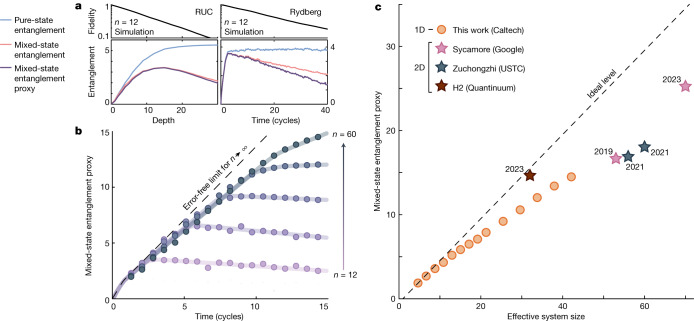
Experimental mixed-state entanglement. **a**, We develop an experimentally measurable proxy that lower-bounds the log negativity, which is a measure of mixed-state entanglement. Here we demonstrate this proxy with error model simulations of RUC and Rydberg evolution. **b**, The experimental mixed-state entanglement proxy; solid lines are guides to the eye. **c**, The maximum entanglement proxy for our experiment can be compared against that of literature examples performing global fidelity estimation with digital quantum processors: Sycamore^2,5^, Zuchongzhi^3,4^ and H2 (ref. ^26^) (text indicates release year). For literature examples, the *x* axis is the number of qubits, whereas for our experiment the effective system size is defined as the number of qubits with the same Hilbert space dimension as our experiment under the Rydberg blockade constraint (Supplementary Information) and is, for instance, roughly 42 at *n* = 60. The data are summarized in Supplementary Table I. 1D, one dimensional; 2D, two dimensional.

We demonstrate the efficacy of ${{\mathcal{E}}}_{P}$ on both noisy RUC evolution and error model simulation of our Rydberg dynamics (Fig. 4a and Supplementary Information). In both cases, the target pure state log negativity increases and saturates, while the exactly calculated mixed-state log negativity reaches a maximum before decaying at late times, behaviour that the entanglement proxy ${{\mathcal{E}}}_{P}$ replicates as a lower bound.

We then plot the experimental entanglement proxy (Fig. 4b), where ${{\mathcal{E}}}_{N}(\left|\psi \right\rangle )$ is extrapolated from small system sizes (Supplementary Fig. 16) and *F* is found from Monte Carlo inference. We observe the entanglement proxy peaks before falling at late times; this peak value increases (Fig. 4c) as a function of effective system size defined as the number of qubits with the same Hilbert space dimension as our experiment under the Rydberg blockade constraint (roughly 42 for *n* = 60).

With equation (2) we can directly compare the results of our present study against RUC evolution in state-of-the-art digital quantum devices^2–5,26^ (Fig. 4c). We find we are within roughly 2 ebits of early tests of quantum advantage^2^ (an ebit is the entanglement of a two-qubit Bell state). For literature examples, we assume targeted states are Haar-random^43,44^, whereas for our experiment we conservatively use the extrapolated log negativity, which is roughly 2 ebits below the expectation for Haar-random states at the largest system sizes (Supplementary Fig. 16).

The mixed-state entanglement proxy ${{\mathcal{E}}}_{P}$ can serve as a useful quality-factor of the ability for different experiments to produce highly entangled states, including for preparation methods besides quench evolution such as quasi-adiabatic ground state preparation (Supplementary Figs. 2 and 23), and could be a more widely applicable alternative to other measures, such as quantum volume^13^, for directing efforts to improve NISQ-era quantum systems.

## The classical cost of quantum simulation

We finally ask: which device, quantum or classical, has a higher fidelity of reproducing a high-entanglement pure target state of interest? Equivalently, in terms of fidelity, what are the minimum classical resources required for a classical computer to outperform the quantum device?

**Fig. 5:**
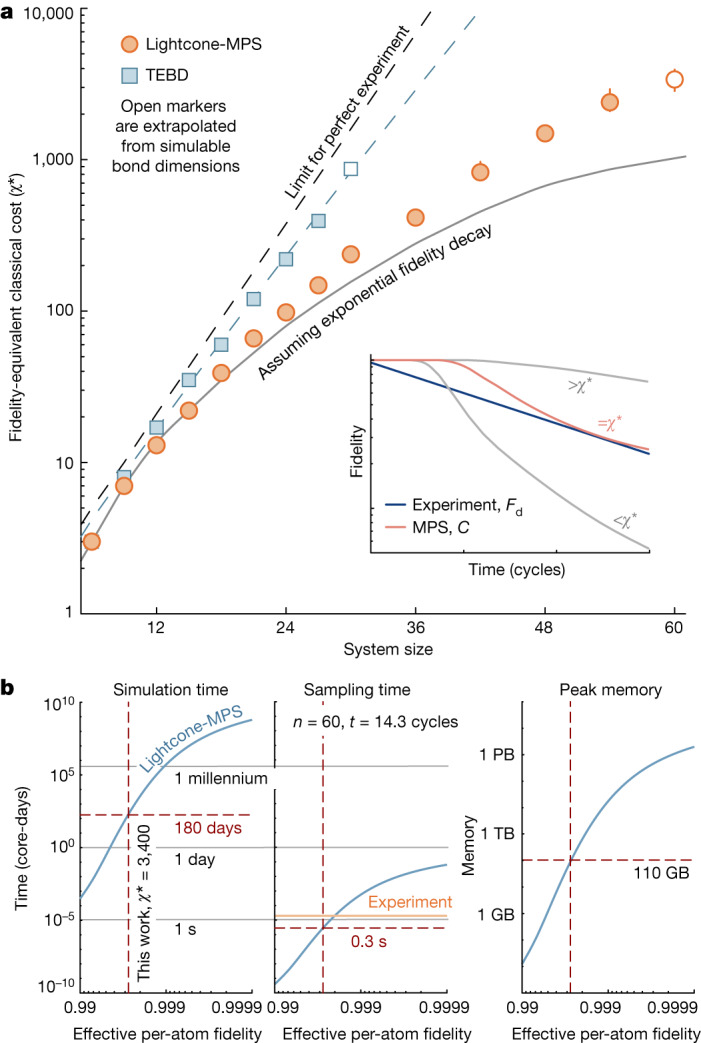
Classical cost to simulate the experiment. **a**, The equivalent classical cost of the experiment, as quantified by the minimum bond dimension, χ*, for the classical simulation to maintain a higher fidelity than the experiment across all times, that is, for *C* > *F*_d_ (inset). We consider several classical algorithms (for example, time-evolving block decimation, TEBD^45^), all of which become impractical at moderate system sizes. This necessitates the introduction of our Lightcone-MPS algorithm (Supplementary Information), which reaches a maximum value of χ* = 3,400 for *n* = 60. **b**, Predicted MPS costs (simulation time, sampling time and peak memory usage) to operate at χ* as a function of the experimental per-atom fidelity (main text). Times are representative of a single 16-core node on the Caltech cluster (Supplementary Information).

To answer this, we compare the fidelity of the experiment against that of the MPS with varying bond dimension. We define the critical bond dimension for a given system size, χ*, as the minimum bond dimension for which the classical fidelity always exceeds the estimated experimental fidelity. This controls the costs of classical simulation: for instance, MPS simulation time scales as ${\mathcal{O}}(n{\chi }^{3})$. We find χ* continually increases as a function of system size (Fig. 5a), reaching a maximum value of χ* = 3,400 for *n* = 60 (Supplementary Fig. 31), and apparently continuing to increase beyond that point.

In performing this study, we used our new Lightcone-MPS algorithm, but considered several alternative approximate classical algorithms, including path integral, matrix product operator, time-dependent variational principle, Schrieffer–Wolff transformation and neural net approaches (Supplementary Information); however, we found the equivalent classical cost of these methods quickly became infeasible, typically well before *n* = 60. As an example, we show χ* for a more conventional MPS approach using time-evolving block decimation^45^ (Fig. 5a).

All calculations used a single 16-core node of the Caltech central computing cluster (Supplementary Information). On this machine, we estimate that running the Lightcone-MPS simulation for *n* = 60 and χ* = 3,400 would entail a peak memory usage of roughly 110 GB (scaling as ${\mathcal{O}}(n{\chi }^{2})$), and would take roughly 11.3 days or 11.3 × 16 ≈ 180 core-days; sampling from the resultant MPS would take roughly 0.3 core-seconds per sample (scaling as ${\mathcal{O}}(n{\chi }^{2})$). For comparison, the experimental cycle time is roughly 1.7 s, limited by array loading and imaging; the actual quantum simulation time is only roughly 1 μs per shot. Just as the classical computer can use several cores, so too can the experiment be parallelized over several atom-array chains simultaneously, which in fact we do already at small system sizes.

We predict these classical costs are highly sensitive to the effective per-atom fidelity, ${\mathcal{F}}$, defined by ${{\mathcal{F}}}^{nt}\equiv F(n,t)$ (Fig. 5b and Supplementary Information). For instance, the simulation time scales as $\approx {(1-{\mathcal{F}})}^{-10}$ around the experimental ${\mathcal{F}}$. Although specialized classical hardware^11,46,47^ may more readily perform the present approximate classical simulations, we thus expect small improvements in the quantum fidelity may soon make the experiment out of reach of even these more advanced classical systems.

## Outlook

As quantum systems tackle tasks of rising complexity, it is increasingly important to understand their ability to produce states in the highly entangled, beyond-classically-exact regime. Here we have studied this regime directly by measuring the global fidelity of an analogue quantum simulator with up to 60 atoms.

A careful analysis (Supplementary Fig. 14) indicates that with reasonable classical resources, our Monte Carlo inference protocol is scalable to an order-of-magnitude larger system sizes than were studied here, potentially enabling fidelity estimation for system sizes with *n* ≈ 500. It is also applicable for digital devices^2–5,26^ that are affected by non-Markovian noises such as control errors^2^, which could then lead to non-exponential scaling of global fidelities in certain parameter regimes. Furthermore, it could be applied to analogue quantum simulators for itinerant particles^15,18,48^. Further, one may imagine applying the same basic technique to cross-platform comparisons^49–51^ between erroneous quantum devices by varying the decoherence of each: a form of zero-noise extrapolation^52^.

Additionally, we have addressed a longstanding problem by introducing a simple proxy of the experimental mixed-state entanglement. This entanglement proxy can serve as a universal quality-factor comparable amongst analogue and digital quantum devices as a guide for improving future systems, and may act as a probe for detecting topological order^53,54^ and measurement-induced criticality^55^.

Finally, we have studied the equivalent classical cost of our experiment on the level of global fidelity, which we note could be greatly increased through the use of erasure conversion^21,56,57^. Similar techniques could be applied to quantify the classical cost of measuring physical observables^9,58^, and to benchmark the performance of approximate classical algorithms themselves through comparison to high fidelity quantum data. Although here we have focused on one-dimensional systems to exploit the power of MPS representations, using higher-dimensional systems^59,60^, while maintaining high fidelities, may prove even more difficult for classical algorithms. We emphasize that in contrast to many previous experiments^2–5^ that explicitly targeted spatiotemporally complex quantum evolution when exploring the limits of classical simulation, here the dynamics we have studied are one-dimensional and both space- and time-independent, yet still begin to reach a regime of classical intractability. Ultimately, our results showcase the present and potential computational power of analogue quantum simulators, encouraging an auspicious future for these platforms^18^.

## Online content

Any methods, additional references, Nature Portfolio reporting summaries, source data, extended data, supplementary information, acknowledgements, peer review information; details of author contributions and competing interests; and statements of data and code availability are available at 10.1038/s41586-024-07173-x.

### Supplementary information

Supplementary Information

## Data Availability

The data supporting this study are available from the corresponding author upon request.

## Code availability

The codes supporting this study are available from the corresponding author upon request.

## References

[1] Preskill J (2018). Quantum computing in the NISQ era and beyond. Quantum.

[2] Arute F (2019). Quantum supremacy using a programmable superconducting processor. Nature.

[3] Wu Y (2021). Strong quantum computational advantage using a superconducting quantum processor. Phys. Rev. Lett..

[4] Zhu Q (2022). Quantum computational advantage via 60-qubit 24-cycle random circuit sampling. Sci. Bull..

[5] Morvan, A. et al. Phase transition in random circuit sampling. Preprint at https://arxiv.org/abs/2304.11119 (2023).

[6] Vidal G, Werner RF (2002). Computable measure of entanglement. Phys. Rev. A.

[7] Preskill, J. Quantum computing and the entanglement frontier. Preprint at https://arxiv.org/abs/1203.5813 (2012).

[8] Ghosh S, Deshpande A, Hangleiter D, Gorshkov AV, Fefferman B (2023). Complexity phase transitions generated by entanglement. Phys. Rev. Lett..

[9] Kechedzhi K (2024). Effective quantum volume, fidelity and computational cost of noisy quantum processing experiments. Future Gener. Comput. Syst..

[10] Bharti K (2022). Noisy intermediate-scale quantum algorithms. Rev. Modern Phys..

[11] Hauru, M. et al. Simulation of quantum physics with tensor processing units: brute-force computation of ground states and time evolution. Preprint at https://arxiv.org/abs/2111.10466 (2021).

[12] Neill C (2018). A blueprint for demonstrating quantum supremacy with superconducting qubits. Science.

[13] Cross AW, Bishop LS, Sheldon S, Nation PD, Gambetta JM (2019). Validating quantum computers using randomized model circuits. Phys. Rev. A.

[14] Choi J (2023). Preparing random states and benchmarking with many-body quantum chaos. Nature.

[15] Mark DK, Choi J, Shaw AL, Endres M, Choi S (2023). Benchmarking quantum simulators using ergodic quantum dynamics. Phys. Rev. Lett..

[16] Proctor T, Rudinger K, Young K, Nielsen E, Blume-Kohout R (2022). Measuring the capabilities of quantum computers. Nat. Phys..

[17] Cirac JI, Zoller P (2012). Goals and opportunities in quantum simulation. Nat. Phys..

[18] Daley AJ (2022). Practical quantum advantage in quantum simulation. Nature.

[19] Browaeys A, Lahaye T (2020). Many-body physics with individually controlled Rydberg atoms. Nat. Phys..

[20] Altman E (2021). Quantum simulators: architectures and opportunities. PRX Quantum.

[21] Scholl P (2023). Erasure conversion in a high-fidelity Rydberg quantum simulator. Nature.

[22] Shaw AL (2023). Dark-state enhanced loading of an optical tweezer array. Phys. Rev. Lett..

[23] Bridgeman JC, Chubb CT (2017). Hand-waving and interpretive dance: an introductory course on tensor networks. J. Phys. A Math. Theor..

[24] Vidal G (2003). Efficient classical simulation of slightly entangled quantum computations. Phys. Rev. Lett..

[25] Zhou Y, Stoudenmire EM, Waintal X (2020). What limits the simulation of quantum computers?. Phys. Rev. X.

[26] Moses, S. A. et al. A race race track trapped-ion quantum processor. *Phys. Rev. X* **13**, 041052 (2023).

[27] Nielsen, M. A. & Chuang, I. L. *Quantum Computation and Quantum Information* (Cambridge Univ. Press, 2010).

[28] Zhang X, Kim E, Mark DK, Choi S, Painter O (2023). A superconducting quantum simulator based on a photonic-bandgap metamaterial. Science.

[29] Haah J, Hastings MB, Kothari R, Low GH (2021). Quantum algorithm for simulating real time evolution of lattice Hamiltonians. SIAM J. Comput..

[30] Tran MC (2019). Locality and digital quantum simulation of power-law interactions. Phys. Rev. X.

[31] Kaufman AM (2016). Quantum thermalization through entanglement in an isolated many-body system. Science.

[32] Dalzell, A. M., Hunter-Jones, N., & Brandão, G. S. L. Random quantum circuits transform local noise into global white noise. Preprint at https://arxiv.org/abs/2111.14907 (2021).

[33] Ganaie MA, Hu M, Malik AK, Tanveer M, Suganthan PN (2022). Ensemble deep learning: a review. Eng. Appl. Artificial Intell..

[34] Islam R (2015). Measuring entanglement entropy in a quantum many-body system. Nature.

[35] Linke NM (2018). Measuring the Rényi entropy of a two-site Fermi-Hubbard model on a trapped ion quantum computer. Phys. Rev. A.

[36] Brydges T (2019). Probing Rényi entanglement entropy via randomized measurements. Science.

[37] Plenio MB (2005). Logarithmic negativity: a full entanglement monotone that is not convex. Phys. Rev. Lett..

[38] O’Donnell, R. & Wright, J. Efficient quantum tomography. In *Proc. Forty-Eighth Annual ACM Symposium on Theory of Computing, STOC ’16* 899–912 (Association for Computing Machinery, 2016).

[39] Haah J, Harrow AW, Ji Z, Wu X, Yu N (2017). Sample-optimal tomography of quantum states. IEEE Trans. Inform. Theory.

[40] Elben A (2020). Mixed-state entanglement from local randomized measurements. Phys. Rev. Lett..

[41] Mooney GJ, White GAL, Hill CD, Hollenberg LCL (2021). Whole-device entanglement in a 65-qubit superconducting quantum computer. Adv. Quant. Technol..

[42] Lee S, Chi DP, Oh SD, Kim J (2003). Convex-roof extended negativity as an entanglement measure for bipartite quantum systems. Phys. Rev. A.

[43] Bhosale UT, Tomsovic S, Lakshminarayan A (2012). Entanglement between two subsystems, the Wigner semicircle and extreme-value statistics. Phys. Rev. A.

[44] Datta A (2010). Negativity of random pure states. Phys. Rev. A.

[45] Vidal G (2004). Efficient simulation of one-dimensional quantum many-body systems. Phys. Rev. Lett..

[46] Ganahl M (2023). Density matrix renormalization group with tensor processing units. PRX Quantum.

[47] Häner, T. & Steiger, D. S. 0.5 Petabyte simulation of a 45-qubit quantum circuit. In *Proc. International Conference for High Performance Computing, Networking, Storage and Analysis, SC ’17* (Association for Computing Machinery, 2017).

[48] Gross C, Bloch I (2017). Quantum simulations with ultracold atoms in optical lattices. Science.

[49] Elben A (2020). Cross-platform verification of intermediate scale quantum devices. Phys. Rev. Lett..

[50] Zhu D (2022). Cross-platform comparison of arbitrary quantum states. Nat. Commun..

[51] Carrasco J, Elben A, Kokail C, Kraus B, Zoller P (2021). Theoretical and experimental perspectives of quantum verification. PRX Quantum.

[52] Li Y, Benjamin SC (2017). Efficient variational quantum simulator incorporating active error minimization. Phys. Rev. X.

[53] Lee YA, Vidal G (2013). Entanglement negativity and topological order. Phys. Rev. A.

[54] Lu T-C, Hsieh TH, Grover T (2020). Detecting topological order at finite temperature using entanglement negativity. Phys. Rev. Lett..

[55] Sang S (2021). Entanglement negativity at measurement-induced criticality. PRX Quantum.

[56] Wu Y, Kolkowitz S, Puri S, Thompson JD (2022). Erasure conversion for fault-tolerant quantum computing in alkaline earth Rydberg atom arrays. Nat. Commun..

[57] Ma S (2023). High-fidelity gates and mid-circuit erasure conversion in an atomic qubit. Nature.

[58] Trivedi, R., Rubio, A. F. & Cirac, J. I. Quantum advantage and stability to errors in analogue quantum simulators. Preprint at https://arxiv.org/abs/2212.04924 (2022). 10.1038/s41467-024-50750-x PMC11297267 39095381

[59] Scholl P (2021). Quantum simulation of 2D antiferromagnets with hundreds of Rydberg atoms. Nature.

[60] Ebadi S (2021). Quantum phases of matter on a 256-atom programmable quantum simulator. Nature.

